# Clinical Significance of Serum (1→3)-β-D-Glucan Positivity in Cryptococcosis: A Retrospective Cohort Study

**DOI:** 10.3390/jof12060427

**Published:** 2026-06-11

**Authors:** Aoi Miyazaki, Shohei Hamada, Hiroko Okabayashi, Kimitaka Akaike, Aiko Masunaga, Shinichiro Okamoto, Yusuke Tomita, Hidenori Ichiyasu, Takuro Sakagami

**Affiliations:** Department of Respiratory Medicine, Kumamoto University Hospital, Faculty of Life Sciences, Kumamoto University, 1-1-1 Honjo, Chuo-ku, Kumamoto 860-8556, Japan; musha_kiyo@outlook.jp (A.M.); aiko.masunaga@me.com (A.M.);

**Keywords:** cryptococcosis, β-D-glucan, disseminated cryptococcosis

## Abstract

Cryptococcosis is associated with negative serum (1→3)-β-D-glucan (BDG) results, but the clinical significance of serum BDG (sBDG) positivity is unclear. We characterized the clinical features of sBDG-positive cryptococcosis in patients with cryptococcal disease. We measured the sBDG levels and classified patients as BDG-positive (>11 pg/mL) or BDG-negative. Clinical characteristics, diagnostic modalities, antifungal treatment, post-treatment BDG level changes, and 90-day cryptococcosis-related mortality were compared. BDG-positive patients showed disseminated cryptococcosis, higher inflammatory marker levels, lower serum albumin levels, higher culture positivity rates, and higher cryptococcal antigen titers than BDG-negative patients. Combination antifungal therapy was administered more frequently in the BDG-positive group; repeat measurements in 15 patients showed a significant decrease in sBDG levels. The 90-day mortality rate was significantly higher in the BDG-positive than in the BDG-negative group (37.5% vs. 3.9%), and overall survival was significantly lower. sBDG levels may be elevated in cases of disseminated or severe disease. Serum BDG positivity may reflect a higher fungal burden, greater disease severity, and poorer short-term outcomes.

## 1. Introduction

Cryptococcosis is a life-threatening invasive fungal infection predominantly caused by *Cryptococcus neoformans* and *Cryptococcus gattii*. This disease primarily affects immunocompromised individuals; however, infections occur in immunocompetent hosts. Severe forms of cryptococcal meningitis and disseminated cryptococcosis are associated with substantial morbidity and mortality, highlighting the importance of timely diagnosis and appropriate clinical assessment [1].

The diagnosis of cryptococcosis relies primarily on fungal culture, histopathological examination, and the detection of cryptococcal capsular polysaccharide antigen (CrAg) [2,3]. Owing to its high sensitivity and specificity in both serum and cerebrospinal fluid (CSF), CrAg testing is the cornerstone diagnostic method recommended by international clinical practice guidelines [4]. Although culture remains essential for definitive diagnosis and species identification, its limited sensitivity and prolonged turnaround time in some settings may delay appropriate treatment [5,6].

(1→3)-β-D-Glucan (BDG) is a polysaccharide component of the fungal cell wall that is widely used as a non-culture-based surrogate marker for invasive fungal infections. Serum BDG (sBDG) testing has demonstrated clinical utility in the diagnosis and management of invasive candidiasis, invasive aspergillosis, and *Pneumocystis jirovecii* pneumonia [7]. However, the role of sBDG testing in cryptococcosis remains unclear. Although sBDG serves as a biomarker of fungal infection in various invasive mycoses, it is not considered as a diagnostic marker for cryptococcosis and is not included in current clinical diagnostic criteria for cryptococcal disease [2].

This exclusion is largely attributed to the unique biological characteristics of *Cryptococcus* species. Unlike many other pathogenic fungi, *Cryptococcus* possesses a thick polysaccharide capsule and contains relatively low amounts of BDG in its cell wall [8,9]. In an early clinical study investigating BDG levels in patients with fungal infections, BDG results were negative in patients with cryptococcosis, suggesting that BDG levels do not increase in cryptococcal disease [10]. Conversely, subsequent studies have suggested that the clinical relevance of BDG in cryptococcosis may vary according to specimen type. For example, the CSF BDG levels were detectable in patients with human immunodeficiency virus (HIV)-associated cryptococcal meningitis and correlated with fungal burden and short-term mortality [11]. These findings suggest that BDG may reflect disease burden within the central nervous system. However, such observations are limited to CSF specimens and cannot be directly extrapolated to sBDG levels.

Regarding the clinical implications of sBDG in cryptococcosis, despite sporadic case reports and autopsy-based studies documenting elevated sBDG levels in patients with cryptococcemia or disseminated disease [11,12], its diagnostic value, prognostic significance, and potential role in disease monitoring have not been systematically evaluated. Moreover, although CrAg often remains positive for prolonged periods after antifungal therapy, CrAg titers are not recommended for routine monitoring of treatment response or relapse [3,13]. We hypothesized that sBDG would provide complementary clinical information beyond that obtained from CrAg testing; however, current guidelines do not recommend BDG for monitoring or prognostic assessment in cryptococcosis [2,3].

Therefore, we aimed to characterize the clinical features of sBDG-positive cryptococcosis and clarify the potential clinical implications of elevated sBDG levels in patients with cryptococcal disease.

## 2. Methods

### 2.1. Participants

We retrospectively reviewed the medical records of patients with cryptococcosis treated at Kumamoto University Hospital between January 2005 and January 2025. Patients were consecutively enrolled according to the following inclusion criteria: (1) a definitive diagnosis of cryptococcosis, and (2) sBDG measurement performed at the time of cryptococcosis diagnosis. The exclusion criteria were as follows: (1) age < 18 years; (2) coexisting fungal infections; (3) no antifungal treatment for cryptococcosis; and (4) absence of follow-up medical records. All clinical and laboratory data were extracted from the patients’ medical records.

Patients were categorized according to the primary diagnostic modality as follows: (1) Microbiological diagnosis, in which *Cryptococcus* was identified by microbiological testing; (2) antigen-based diagnosis, in which *Cryptococcus* was not detected by microbiological testing but the CrAg test was positive; and (3) pathological diagnosis, in which *Cryptococcus* was not detected by microbiological testing and CrAg testing was negative, but *Cryptococcus* was identified by histopathological examination.

Patients were classified according to the site of infection as having pulmonary cryptococcosis, defined as cryptococcal infection confined to the lungs, or disseminated cryptococcosis, defined as infection confirmed by positive cultures from sterile sites (e.g., blood or CSF) or a positive CrAg test result in the CSF.

The study was conducted in accordance with the principles of the Declaration of Helsinki. The Institutional Review Board of Kumamoto University Hospital approved the study (approval number: 3114) and waived the requirement for written informed consent because of its retrospective design.

### 2.2. Measurement of sBDG Concentration and CrAg in Serum

sBDG levels were measured using the WAKO BDG test (Wako Pure Chemical Industries, Ltd., Tokyo, Japan). sBDG positivity was defined using a cut-off value of >11 pg/mL for invasive fungal infections [14]. No predefined criteria were set for the timing of sBDG measurement, which was performed at the discretion of the attending physicians. CrAg testing was performed using a semiquantitative latex agglutination assay for *C. neoformans* (BML Inc., Tokyo, Japan).

### 2.3. Definition of Clinical Indices

Cryptococcosis was diagnosed based on at least one of the following criteria: (1) microbiological identification of *Cryptococcus* by culture or India ink staining; (2) histopathological demonstration of *Cryptococcus* in tissue specimens; or (3) a positive CrAg test result in the serum, CSF, or bronchoalveolar lavage fluid.

Cryptococcosis-related mortality was defined as death occurring in patients with unresolved clinical manifestations who were still receiving antifungal treatment. Patients who were lost to follow-up or withdrew from the study for reasons unrelated to cryptococcosis were excluded from the mortality analysis.

Changes in the sBDG levels in response to antifungal treatment were evaluated. In cases where post-treatment sBDG measurements were unavailable because of death or censoring, the last available sBDG value was used for comparison.

### 2.4. Statistical Analysis

Continuous variables were expressed as medians with interquartile ranges (IQRs) and compared using the Mann–Whitney U test. Categorical variables were compared using Fisher’s exact test. Survival analysis for cryptococcosis-related mortality was performed using the Kaplan–Meier method and compared using the log-rank test. Statistical significance was defined as a two-sided *p*-value < 0.05. Multivariable analysis was not performed because of the small number of events. All statistical analyses were conducted using SPSS software (version 20.0; IBM Corp., Armonk, NY, USA).

## 3. Results

### 3.1. Study Population and Baseline Characteristics at Diagnosis

**Figure 1:**
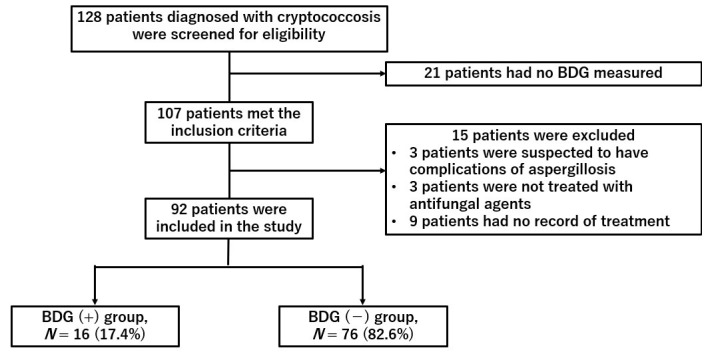
Flow diagram showing patient inclusion and exclusion criteria for this study.

Between January 2005 and January 2025, 128 patients diagnosed with cryptococcosis and hospitalized at our institution were screened for eligibility. Of these, 107 patients who underwent sBDG measurement at the time of diagnosis met the inclusion criteria. Three patients with suspected concomitant aspergillosis, three who did not receive antifungal therapy, and nine with insufficient clinical data regarding their treatment course were excluded. Consequently, 92 patients were included in the final analysis (Figure 1).

Among the included patients, 16 (17.4%) had sBDG levels exceeding the predefined cut-off value (WAKO assay > 11 pg/mL) at diagnosis and were classified into the BDG-positive group (BDG [+]); the remaining 76 patients (82.6%) were classified into the BDG-negative group (BDG [−]). The baseline characteristics of the study population are summarized in Table 1. The median age of the overall cohort was 68 years, and 48 patients (52.2%) were male. Only one patient had AIDS. No significant differences were observed between the BDG (+) and BDG (−) groups with respect to age, sex, or history of immunosuppressive conditions, including systemic corticosteroid use, immunosuppressive therapy, or anticancer chemotherapy.

Laboratory findings at diagnosis revealed significant differences between the two groups. Patients in the BDG (+) group had significantly higher white blood cell counts than those in the BDG (−) group (median values; *p* = 0.020). C-reactive protein levels were also significantly higher in the BDG (+) group (*p* < 0.001), whereas lymphocyte counts and serum albumin levels were significantly lower (*p* = 0.009 and *p* = 0.005, respectively).

Regarding disease classification, 62 patients (67.4%) were diagnosed with pulmonary cryptococcosis, 21 (22.8%) with disseminated cryptococcosis, and 9 (9.8%) with other forms of cryptococcosis. The proportion of patients with pulmonary cryptococcosis was significantly lower in the BDG (+) group than in the BDG (−) group (18.8% vs. 77.6%, *p* < 0.05). In contrast, disseminated cryptococcosis was significantly more common in the BDG (+) group than in the BDG (−) group (68.8% vs. 13.2%, *p* < 0.05).

### 3.2. Diagnostic Methods and CrAg Burden

**Figure 2:**
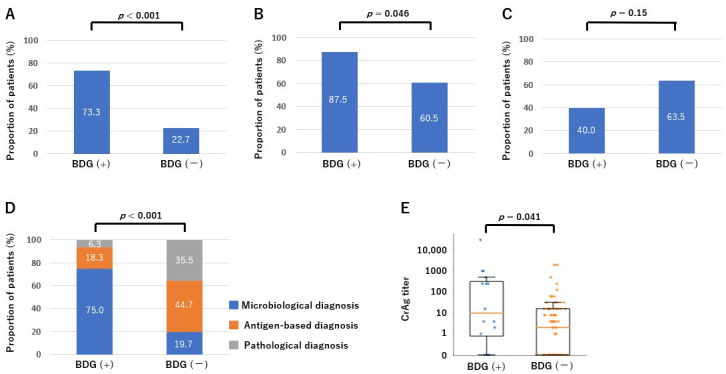
Comparison of the diagnostic test results between β-D-glucan (BDG)-positive (+) and BDG-negative (−) groups. Bar graphs showing (**A**) culture positivity rate, (**B**) cryptococcal antigen positivity rate, (**C**) histopathological positivity rate, and (**D**) diagnostic method. (**E**) Box-and-whisker plots showing cryptococcal antigen titers as individual data points.

When diagnostic modalities were compared, *Cryptococcus* species were isolated by culture in 73.3% of patients in the BDG (+) group and 22.7% of those in the BDG (−) group. This difference was significant (*p* < 0.001; Figure 2A). The positivity rate of serum or CSF CrAg testing was higher in the BDG (+) group than in the BDG (−) group (87.5% vs. 60.5%, respectively; *p* = 0.046; Figure 2B).

In contrast, the proportion of patients diagnosed by histopathological examination was higher in the BDG (−) group (63.5%) than in the BDG (+) group (40.0%), although this difference did not reach significance (*p* = 0.15; Figure 2C). An overall comparison of the distribution of diagnostic modalities demonstrated a significant difference between the BDG (+) and BDG (−) groups (*p* < 0.001; Figure 2D).

In addition, the median CrAg titer at diagnosis was significantly higher in the BDG (+) group than in the BDG (−) group (10 vs. 2, respectively; *p* = 0.041; Table 1 and Figure 2E).

### 3.3. Antifungal Treatment and Changes in sBDG Levels

**Figure 3:**
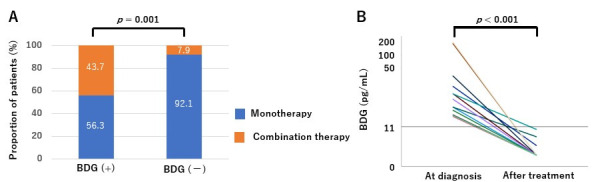
Comparison of antifungal therapy and changes in BDG levels between the BDG (+) and BDG (−) groups. (**A**) Bar graph of antifungal treatment differences between the groups. (**B**) Changes in serum BDG levels before and after treatment for each individual patient.

Combination antifungal therapy, defined as the use of two or more antifungal agents as initial treatment, was administered significantly more frequently in the BDG (+) group than in the BDG (−) group (43.8% vs. 7.9%, *p* < 0.001; Figure 3A). Details of the treatment regimens are presented in Table S1. Among the 16 patients in the BDG (+) group, 15 underwent repeat BDG measurements after antifungal therapy initiation. In all patients, the post-treatment sBDG levels were significantly lower than the baseline values (*p* < 0.001; Figure 3B). This decline in sBDG levels was observed in both survivors and non-survivors.

### 3.4. Survival Prognosis

**Figure 4:**
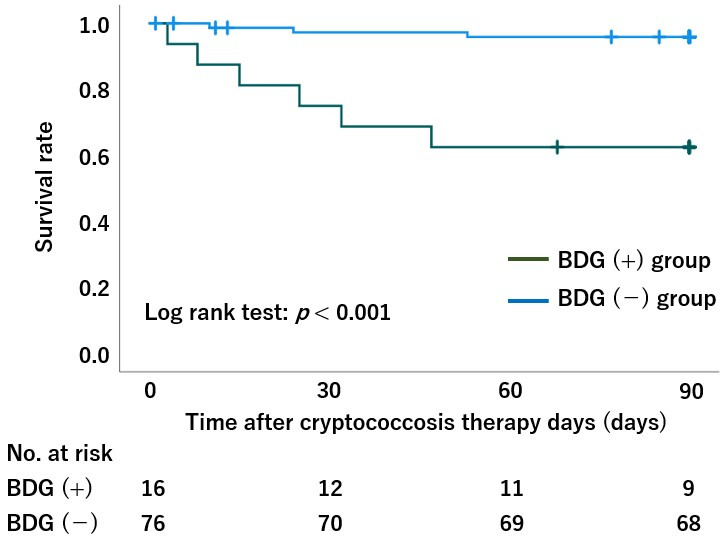
Kaplan–Meier survival curves comparing the 90-day cryptococcosis-related mortality rates between the BDG (+) and BDG (−) groups.

The overall 90-day cryptococcosis-related mortality rate of the entire cohort was 9.8% (9 out of 92 patients). Six out of 16 patients (37.5%) in the BDG (+) group died within 90 days, whereas only 3 out of 76 patients (3.9%) in the BDG (−) group died during the same period, indicating a significantly higher mortality rate in the BDG (+) group. Kaplan–Meier survival analysis demonstrated significantly lower survival in the BDG (+) group than in the BDG (−) group. This difference was confirmed using the log-rank test, which showed a significant difference between the two survival curves (*p* < 0.001; Figure 4).

## 4. Discussion

In this study, we provide the first comprehensive analysis of sBDG dynamics in a large cohort of patients with predominantly HIV-negative cryptococcosis. We demonstrated that 17.4% of patients had positive sBDG results at diagnosis and sBDG positivity was associated with a markedly higher 90-day cryptococcosis-related mortality rate than sBDG negativity (37.5% vs. 3.9%). Although sBDG was not identified as an independent prognostic factor, largely because of the limited number of fatal cases precluding a robust multivariable analysis, our findings suggest that elevated sBDG reflects greater disease severity or fungal burden and is associated with adverse clinical outcomes. Thus, sBDG may serve as a useful adjunctive marker for assessing disease severity in cryptococcosis rather than as a replacement for established diagnostic or prognostic tools.

Traditionally, BDG levels have been considered negative in cryptococcosis. In 1995, Miyazaki et al. reported that none of the 10 patients with cryptococcosis exhibited elevated BDG levels despite positive CrAg test results [10]. Accordingly, current diagnostic frameworks for invasive fungal diseases do not recognize BDG as a diagnostic marker for cryptococcosis [2,3]. However, more recent reports describing positive sBDG results in patients with cryptococcosis suggest that this assumption is not universally applicable [11,12]. Our findings indicate that sBDG levels may be elevated in a subset of patients with cryptococcosis, particularly those with severe disease, challenging the traditional assumption that BDG levels do not increase in cryptococcal infection.

The biological basis for the low detectability of BDG in cryptococcosis lies in the unique composition of the cryptococcal cell wall. Unlike most pathogenic fungi, in which (1→3)-BDG is a major structural component, *Cryptococcus* species possess a cell wall characterized by low (1→3)-BDG content and a predominance of an α-glucan-rich capsule and (1→6)-BDG [9]. This structural feature helps explain why BDG assays often yield negative results in cryptococcal infections. Therefore, a sufficiently high fungal burden may be required to release detectable quantities of circulating (1→3)-BDG. Supporting this hypothesis, a study of HIV-associated cryptococcal meningitis showed that BDG is frequently detectable in the CSF, particularly in cases with a high fungal burden, and CSF BDG levels strongly correlate with fungal load and CrAg titers [11]. Although sBDG appears to be less sensitive than CSF BDG, these findings indicate that *Cryptococcus* can release measurable amounts of BDG when the organism burden is high. Moreover, previous studies reporting negative BDG results primarily included patients with localized pulmonary cryptococcosis [10], whereas reports describing BDG positivity mainly involved severe disease, such as cryptococcal meningitis [11,12]. These observations further support the hypothesis that BDG positivity is associated with fungal burden in cryptococcosis. In our study, sBDG positivity was uncommon among patients with localized pulmonary cryptococcosis (4.8%), whereas more than half of the patients with disseminated cryptococcosis had positive sBDG results, consistent with previous results and our proposed hypothesis.

CSF BDG levels decline rapidly and return to normal following effective antifungal therapy [11], whereas CrAg levels decline more slowly and may remain positive after treatment in some patients [15]. Similarly, we observed consistent declines in sBDG levels after treatment among patients in the BDG (+) group. Although the relationship between sBDG kinetics and clinical outcomes has not been extensively investigated in cryptococcosis, studies of other invasive fungal infections have suggested potential clinical relevance. For example, Carelli et al. demonstrated that a pronounced decline in serial sBDG measurements (“BDG downslope”) was associated with improved survival in invasive candidiasis [16]. These findings suggest that BDG may function as a dynamic marker of fungal burden or treatment response. In our study, however, all fatal cases exhibited declining sBDG levels following antifungal therapy, indicating that BDG clearance does not necessarily equate to microbiological eradication or clinical cure. Given the inherently low production of BDG by *Cryptococcus* spp., BDG may become undetectable despite persistent infection, and its interpretation should therefore be approached with caution.

Our study clarified the relationship between sBDG positivity and clinical outcomes. Patients in the BDG-positive group exhibited features of more severe illness, including higher inflammatory marker levels, lower serum albumin levels, and a higher prevalence of disseminated disease, all of which likely contributed to their poorer prognosis. In contrast, sBDG positivity has not been consistently associated with clinical outcomes in invasive aspergillosis, invasive candidiasis, or *P. jirovecii* pneumonia [17]. This difference may be attributable to the limited elevation of BDG levels in cryptococcosis and the observation that BDG positivity appears to reflect a higher fungal burden. Therefore, BDG may have distinct clinical significance in cryptococcosis compared with other invasive fungal infections.

The disparity in BDG positivity between pulmonary and disseminated cryptococcosis parallels the well-established prognostic differences between these disease forms. Isolated pulmonary cryptococcosis generally has a favorable prognosis, with a reported mortality rate of <1%, whereas disseminated disease, particularly when involving the central nervous system, is associated with mortality rates approaching 50% [18]. Consistent with these findings, pulmonary cryptococcosis in our cohort was associated with low sBDG positivity and favorable outcomes, whereas disseminated disease was associated with higher sBDG positivity and poorer prognosis. Even among microbiologically confirmed cases, sBDG positivity was associated with increased mortality. Collectively, these findings support the interpretation that BDG positivity in cryptococcosis reflects a high fungal burden and severe disease and may therefore serve as a warning sign for poor clinical outcomes.

From a diagnostic perspective, cryptococcosis is most reliably diagnosed by fungal culture, histopathological examination, or the detection of CrAg [2,3]. In both pulmonary cryptococcosis and cryptococcal meningitis, fungal culture has limitations related to sensitivity and turnaround time, whereas CrAg testing is rapid and highly sensitive [5,6,19]. Accordingly, the current guidelines recommend CrAg testing as the cornerstone of cryptococcal diagnosis [3,4]. However, CrAg testing is generally performed only when cryptococcosis is clinically suspected. In contrast, sBDG testing is widely used as a broad screening tool for invasive fungal infections [20,21]. Our findings suggest that unexplained sBDG positivity, particularly in patients with risk factors for severe fungal disease, should prompt the consideration of cryptococcosis even when it is not initially suspected. Failure to recognize this possibility may result in misdiagnosis or inappropriate empirical therapy, such as echinocandin administration, which lacks activity against *Cryptococcus*. Delays in diagnosis and initiation of appropriate antifungal therapy are associated with worse outcomes in cryptococcosis [22,23], whereas early treatment improves survival [24,25].

This study has some limitations. As a single-center retrospective analysis, in which most patients were HIV-negative, the generalizability of the findings may be limited. Additionally, baseline prediagnostic sBDG levels were unavailable. Nevertheless, the consistent decline in the sBDG levels following treatment and absence of concurrent invasive fungal infections support our conclusion that elevated sBDG levels were attributable to cryptococcosis. Finally, the limited number of fatal cases precluded a robust multivariable analysis of mortality predictors.

## 5. Conclusions

We demonstrated that sBDG levels may be elevated in a subset of patients with cryptococcosis, particularly those with disseminated or severe disease, challenging the traditional view that BDG is uniformly negative in cryptococcal infections. Therefore, clinicians should consider cryptococcosis in the differential diagnosis of unexplained sBDG positivity to avoid diagnostic delays and inappropriate antifungal therapy.

## Figures and Tables

**Table 1:** Characteristics of the study participants.

Item	All *N* = 92	BDG (+) Group *N* = 16	BDG (−) Group *N* = 76	*p*-Value
Age (years)	68 (59.3–73.0)	68 (54.5–75.8)	68 (60.3–73.0)	0.87
Male, sex	48 (52.2%)	8 (50.0%)	40 (52.6%)	0.85
*Cryptococcus* organism				0.42
*C. neoformans*	25/26 (96.2%)	10/11 (90.9%)	15/15 (100%)	
*C. gattii*	1/26 (3.8%)	1/11 (9.1%)	0/15 (0%)	
Comorbidities				
Diabetes	19 (20.7%)	5 (31.3%)	14 (18.4%)	0.31
Hepatic cirrhosis	6 (6.5%)	1 (6.3%)	5 (6.6%)	1.00
CTD	31 (33.7%)	9 (56.3%)	22 (28.9%)	0.036
AIDS	1 (1.1%)	1 (6.3%)	0 (0.0%)	0.174
Solid tumors	25 (27.2%)	3 (18.8%)	22 (28.9%)	0.54
Hematologic malignancy	19 (20.7%)	2 (12.5%)	17 (22.4%)	0.51
Medical history				
Corticosteroids	33 (35.9%)	9 (56.3%)	24 (31.6%)	0.061
Immunosuppressants	19 (20.7%)	4 (25.0%)	15 (19.7%)	0.74
Chemotherapy	15 (16.3%)	0 (0.0%)	15 (19.7%)	0.065
Laboratory findings				
White blood cells (cells/μL)	6650 (5100–9175)	10,200 (6800–14,100)	6100 (4775–8375)	0.020
Lymphocyte count (cells/μL)	1210 (760–1799)	725 (436–1253)	1279 (901–1957)	0.009
Albumin (mg/dL)	3.65 (2.88–4.20)	2.85 (2.10–3.70)	3.75 (3.10–4.20)	0.005
LDH (U/L)	208 (174–281)	245 (197–299)	205 (173–279)	0.077
Creatinine (mg/dL)	0.79 (0.54–1.01)	0.73 (0.52–3.00)	0.80 (0.56–0.99)	0.93
CRP (mg/dL)	0.56 (0.08–4.70)	4.05 (1.07–11.60)	0.27 (0.06–3.44)	<0.001
Cryptococcal antigen titer	4 (0–16)	10 (0–448)	2 (0–16)	0.041
Type of cryptococcosis				<0.001
Disseminated	21 (22.8)	11(68.7)	10 (13.2)	
Pulmonary	62 (67.4)	3 (18.8)	59 (77.6)	
Other	9 (9.8)	2 (12.5)	7 (9.2)	

Data are presented as medians (ranges) or *N* (%). BDG, β-D-glucan; CTD, connective tissue disease; LDH, lactate dehydrogenase; CRP, C-reactive protein.

## Data Availability

The data are not publicly available due to privacy and ethical restrictions but are available from the corresponding author upon reasonable request.

## Supplementary Materials

The following supporting information can be downloaded at: https://www.mdpi.com/article/10.3390/jof12060427/s1, Table S1: Details of the antifungal treatment regimens.

## Abbreviations

The following abbreviations are used in this manuscript:

BDG	(1→3)-β-D-Glucan
CrAg	Cryptococcal capsular polysaccharide antigen
CSF	Cerebrospinal fluid
HIV	Human immunodeficiency virus
IQRs	Interquartile ranges
sBDG	Serum BDG

## References

[1] Rajasingham R., Govender N.P., Jordan A., Loyse A., Shroufi A., Denning D.W., Meya D.B., Chiller T.M., Boulware D.R. (2022). The Global Burden of HIV-Associated Cryptococcal Infection in Adults in 2020: A Modelling Analysis. Lancet Infect. Dis..

[2] Donnelly J.P., Chen S.C., Kauffman C.A., Steinbach W.J., Baddley J.W., Verweij P.E., Clancy C.J., Wingard J.R., Lockhart S.R., Groll A.H. (2020). Revision and Update of the Consensus Definitions of Invasive Fungal Disease from the European Organization for Research and Treatment of Cancer and the Mycoses Study Group Education and Research Consortium. Clin. Infect. Dis..

[3] Perfect J.R., Dismukes W.E., Dromer F., Goldman D.L., Graybill J.R., Hamill R.J., Harrison T.S., Larsen R.A., Lortholary O., Nguyen M.H. (2010). Clinical Practice Guidelines for the Management of Cryptococcal Disease: 2010 Update by the Infectious Diseases Society of America. Clin. Infect. Dis..

[4] World Health Organization (2018). Guidelines for the Diagnosis, Prevention, and Management of Cryptococcal Disease in HIV-Infected Adults, Adolescents and Children, March 2018: Supplement to the 2016 Consolidated Guidelines of the Use of Antiretroviral Drugs for Treating and Preventing HIV Infection.

[5] Temfack E., Rim J.J.B., Spijker R., Loyse A., Chiller T., Pappas P.G., Perfect J., Sorell T.C., Harrison T.S., Cohen J.F. (2021). Cryptococcal Antigen in Serum and Cerebrospinal Fluid for Detecting Cryptococcal Meningitis in Adults Living with Human Immunodeficiency Virus: Systematic Review and Meta-analysis of Diagnostic Test Accuracy Studies. Clin. Infect. Dis..

[6] Zhou Y., Lin P.C., Ye J.R., Su S.S., Dong L., Wu Q., Xu H.Y., Xie Y.P., Li Y.P. (2018). The Performance of Serum Cryptococcal Capsular Polysaccharide Antigen Test, Histopathology and Culture of the Lung Tissue for Diagnosis of Pulmonary Cryptococcosis in Patients Without HIV Infection. Infect. Drug Resist..

[7] Ostrosky-Zeichner L., Alexander B.D., Kett D.H., Vazquez J., Pappas P.G., Saeki F., Ketchum P.A., Wingard J., Schiff R., Tamura H. (2005). Multicenter Clinical Evaluation of the (1→3) β-D-glucan Assay as an Aid to Diagnosis of Fungal Infections in Humans. Clin. Infect. Dis..

[8] Odabasi Z., Paetznick V.L., Rodriguez J.R., Chen E., McGinnis M.R., Ostrosky-Zeichner L. (2006). Differences in Beta-Glucan Levels in Culture Supernatants of a Variety of Fungi. Med. Mycol..

[9] Garcia-Rubio R., de Oliveira H.C., Rivera J., Trevijano-Contador N. (2020). The Fungal Cell Wall: Candida, Cryptococcus, and Aspergillus species. Front. Microbiol..

[10] Miyazaki T., Kohno S., Mitsutake K., Maesaki S., Tanaka K., Ishikawa N., Hara K. (1995). Plasma (1→3)-β-D-Glucan and Fungal Antigenemia in Patients with Candidemia, Aspergillosis, and Cryptococcosis. J. Clin. Microbiol..

[11] Rhein J., Bahr N.C., Morawski B.M., Schutz C., Zhang Y., Finkelman M., Meya D.B., Meintjes G., Boulware D.R. (2014). Detection of High Cerebrospinal Fluid Levels of (1→3)-β-D-Glucan in Cryptococcal Meningitis. Open Forum Infect. Dis..

[12] Obayashi T., Negishi K., Suzuki T., Funata N. (2008). Reappraisal of the Serum (1→3)-β-D-Glucan Assay for the Diagnosis of Invasive Fungal Infections—A Study Based on Autopsy Cases from 6 Years. Clin. Infect. Dis..

[13] hang C.C., Harrison T.S., Bicanic T.A., Chayakulkeeree M., Sorrell T.C., Warris A., Hagen F., Spec A., Oladele R., Govender N.P. (2024). Global Guideline for the Diagnosis and Management of Cryptococcosis: An Initiative of the ECMM and ISHAM in Cooperation with the ASM. Lancet Infect. Dis..

[14] He S., Hang J.P., Zhang L., Wang F., Zhang D.C., Gong F.H. (2015). A Systematic Review and Meta-analysis of Diagnostic Accuracy of Serum 1, 3-β-D-Glucan for Invasive Fungal Infection: Focus on Cutoff Levels. J. Microbiol. Immunol. Infect..

[15] Bennett J.E., Williamson P.R. (2024). Antigen Titers in Cryptococcal Meningitis: What Determines How Fast They Fall?. J. Infect. Dis..

[16] Carelli S., Posteraro B., Torelli R., De Carolis E., Vallecoccia M.S., Xhemalaj R., Cutuli S.L., Tanzarella E.S., Dell’Anna A.M., Lombardi G. (2024). Prognostic Value of Serial (1,3)-β-D-Glucan Measurements in ICU Patients with Invasive Candidiasis. Crit. Care.

[17] Koo S., Baden L.R., Marty F.M. (2012). Post-diagnostic Kinetics of the (1→3)-β-D-Glucan Assay in Invasive Aspergillosis, Invasive Candidiasis, and *Pneumocystis jirovecii* Pneumonia. Clin. Microbiol. Infect..

[18] Tao Z., Pu Q., Shen Y., Zhang S., Wang C., Hu Z., Jin Y., Zhu X., Weng Y. (2024). Clinical Characteristics and Prognostic Factors of Pulmonary and Extrapulmonary Cryptococcosis. BMC Infect. Dis..

[19] Fisher J.F., Valencia-Rey P.A., Davis W.B. (2016). Pulmonary Cryptococcosis in the Immunocompetent Patient—Many Questions, Some Answers. Open Forum Infect. Dis..

[20] Odabasi Z., Mattiuzzi G., Estey E., Kantarjian H., Saeki F., Ridge R.J., Ketchum P.A., Finkelman M.A., Rex J.H., Ostrosky-Zeichner L. (2004). β-D-Glucan as a Diagnostic Adjunct for Invasive Fungal Infections: Validation, Cutoff Development, and Performance in Patients with Acute Myelogenous Leukemia and Myelodysplastic Syndrome. Clin. Infect. Dis..

[21] Farhour Z., Mehraj V., Chen J., Ramendra R., Lu H., Routy J.P. (2018). Use of (1→3)-β-d-Glucan for Diagnosis and Management of Invasive Mycoses in HIV-Infected Patients. Mycoses.

[22] Link A., Okwir M., Nabongo B., Meya D., Iribarren S., Bohjanen P., Kasprzyk D. (2022). Delays in Cryptococcal Meningitis Diagnosis and Care: A Mixed Methods Study in Rural Uganda. Ann. Glob. Health.

[23] Meya D.B., Williamson P.R. (2024). Cryptococcal Disease in Diverse Hosts. N. Engl. J. Med..

[24] Butler E.K., Boulware D.R., Bohjanen P.R., Meya D.B. (2012). Long Term 5-Year Survival of Persons with Cryptococcal Meningitis or Asymptomatic Subclinical Antigenemia in Uganda. PLoS ONE.

[25] Rajasingham R., Meya D.B., Greene G.S., Jordan A., Nakawuka M., Chiller T.M., Boulware D.R., Larson B.A. (2019). Evaluation of a National Cryptococcal Antigen Screening Program for HIV-Infected Patients in Uganda: A Cost-Effectiveness Modeling Analysis. PLoS ONE.

