# Supplementary material for: Clinical Significance of Serum (1→3)-β-D-Glucan Positivity in Cryptococcosis: A Retrospective Cohort Study
Source: J Fungi (Basel). 2026 Jun 11;12(6):427. doi: 10.3390/jof12060427 (PMC13302220; doi:10.3390/jof12060427)
Supplement: Supplementary file 1 [file jof-12-00427-s001.zip › jof-4345717-supplementary.pdf]

Table S1. Details of the antifungal treatment regimens.

Treatment regimen	All<br>N=92
Monotherapy	
Fluconazole	64
Itraconazole	4
Voriconazole	3
Liposomal amphotericin B	7
Amphotericin B	1
Combination therapy	
Liposomal amphotericin B and flucytosine	6
Liposomal amphotericin B and fluconazole	2
Liposomal amphotericin B, voriconazole and flucytosine	1
Amphotericin B and flucytosine	1
Amphotericin B and fluconazole	1
Amphotericin, fluconazole and flucytosine	1
Voriconazole and flucytosine	1

Data are presented as numbers.
